# Extracorporeal membrane oxygenation for severe acute respiratory distress syndrome in adult patients: a systematic review and meta-analysis

**DOI:** 10.5935/0103-507X.20190077

**Published:** 2019

**Authors:** Pedro Vitale Mendes, Livia Maria Garcia Melro, Ho Yeh Li, Daniel Joelsons, Rogerio Zigaib, José Mauro da Fonseca Pestana Ribeiro, Bruno Adler Maccagnan Pinheiro Besen, Marcelo Park

**Affiliations:** 1 Unidade de Terapia Intensiva, Hospital das Clínicas, Faculdade de Medicina, Universidade de São Paulo - São Paulo (SP), Brasil.

**Keywords:** Respiratory insufficiency, Extracorporeal membrane oxygenation, Respiratory distress syndrome, adult, Intensive care units, Meta-analysis

## Abstract

**Objective:**

The evidence of improved survival with the use of extracorporeal membrane oxygenation (ECMO) in acute respiratory distress syndrome is still uncertain.

**Methods:**

This systematic review and meta-analysis was registered in the PROSPERO database with the number CRD-42018098618. We performed a structured search of Medline, Lilacs, and ScienceDirect for randomized controlled trials evaluating the use of ECMO associated with (ultra)protective mechanical ventilation for severe acute respiratory failure in adult patients. We used the Cochrane risk of bias tool to evaluate the quality of the evidence. Our primary objective was to evaluate the effect of ECMO on the last reported mortality. Secondary outcomes were treatment failure, hospital length of stay and the need for renal replacement therapy in both groups.

**Results:**

Two randomized controlled studies were included in the meta-analysis, comprising 429 patients, of whom 214 were supported with ECMO. The most common reason for acute respiratory failure was pneumonia (60% - 65%). Respiratory ECMO support was associated with a reduction in last reported mortality and treatment failure with risk ratios (RR: 0.76; 95%CI 0.61 - 0.95 and RR: 0.68; 95%CI 0.55 - 0.85, respectively). Extracorporeal membrane oxygenation reduced the need for renal replacement therapy, with a RR of 0.88 (95%CI 0.77 - 0.99). Intensive care unit and hospital lengths of stay were longer in ECMO-supported patients, with an additional P50^th^ 14.84 (P25^th^ - P75^th^: 12.49 - 17.18) and P50^th^ 29.80 (P25^th^ - P75^th^: 26.04 - 33.56] days, respectively.

**Conclusion:**

Respiratory ECMO support in severe acute respiratory distress syndrome patients is associated with a reduced mortality rate and a reduced need for renal replacement therapy but a substantial increase in the lengths of stay in the intensive care unit and hospital. Our results may help bedside decision-making regarding ECMO initiation in patients with severe respiratory distress syndrome.

## INTRODUCTION

Veno-venous extracorporeal membrane oxygenation (ECMO) is a modality of respiratory support that allows the maintenance of protective ventilation during the acute phase of the underlying pulmonary disease. In the past 20 years, several case series and observational trials have shown favorable outcomes and significant reductions in mortality rates with the use of ECMO support in patients with acute respiratory distress syndrome (ARDS).^(1-5)^

In 2009, the routine use of ECMO support in selected severe ARDS patients referred to a specialized center was considered cost-effective in a randomized controlled trial published in the UK.^(6)^ Although these results were very promising, the transfer of patients randomized to the ECMO group to a specialized referral center, as well as the absence of a standardized protocol for protective ventilation in the control group, raised concerns regarding the external validity of their results. Furthermore, meta-analyses and systematic reviews published after the publication of that trial have shown conflicting results.^(7,8)^

More recently, the ECMO to Rescue Lung Injury in Severe ARDS (EOLIA) trial showed no survival improvement with the use of ECMO support in ARDS patients.^(9)^ However, a high rate of crossover between groups and an early stop for futility raised the question of whether there is still room for ECMO support in ARDS patients.

In this new scenario, we planned this systematic review and meta-analysis to evaluate whether respiratory ECMO support can improve patient survival or reduce treatment failure in severe ARDS patients in comparison to conventional mechanical ventilation without ECMO. Additionally, we investigated the impact of respiratory ECMO support on the need for renal replacement therapy (RRT) and on intensive care unit (ICU) and hospital length of stay.

## METHODS

### Inclusion and exclusion criteria

Only randomized controlled trials in adult patients with ARDS evaluating the use of ECMO support plus protective mechanical ventilation in comparison to protective mechanical ventilation alone were included. Trials published before the routine use of protective mechanical ventilation such that the ventilatory support was not protective in both the ECMO and control groups were excluded. Pediatric, neonatal and experimental data, as well as observational trials, case series and case reports were excluded.

### Search strategy

Publications from 1966 to July 2018 were included. We searched MEDLINE, LILACS, and ScienceDirect to identify studies evaluating the use of ECMO in patients with acute severe respiratory failure. There was no language restriction. We divided our question into two search blocks to improve the sensitivity. The first block pertained to the ECMO technique and used the following MeSH terms: extracorporeal membrane oxygenation and extracorporeal oxygenation. The second block pertained to our desired population and used the following MeSH terms in Medline: acute respiratory distress syndrome; acute respiratory failure; shock lung.

The resulting outputs were then combined. Duplicate results were excluded. Animal, pediatric, and neonatal studies; case-control studies; case reports; and review articles were then excluded. The remaining articles were evaluated independently by two investigators for eligibility. Disagreements between the investigators were resolved by a third investigator. We also searched personal records and the references of retrieved articles for other potential studies.

### Study evaluation and data extraction

Two investigators independently classified randomized studies included in the meta-analysis with the Cochrane risk of bias tool (Table 1S of the supplementary material).^(10,11)^ A third investigator solved disagreements. Two authors extracted the data. The last reported mortality in each study was collected. We also collected study inclusion and exclusion criteria, patient demographic features (including illness severity), the need for RRT, mechanical ventilation information, and ICU and hospital lengths of stay (LOS). When additional information from the retrieved studies was necessary, an e-mail was sent to the main author requesting the data.

### Statistical analysis

Our primary endpoint was the last reported mortality in the ECMO group in comparison with the conventional mechanical ventilation group.^(12)^ Considering that a high crossover rate was expected in the retrieved trials, we planned additional analyses of treatment failure and per protocol as secondary endpoints. We defined treatment failure as death or crossover in the control group and as death in the ECMO group. The need for renal replacement therapy, as well as ICU and hospital LOS were also evaluated. Heterogeneity among studies was assessed with Cochran's Q statistic and Higgin's I^2^ statistic. Both a p < 0.10 and an I^2^ > 50% were considered suggestive of significant heterogeneity.^(13)^ Due to the special characteristics of the targeted patients and support methodology, we expected that studies would present with low heterogeneity. Thus, a fixed-effects model using the Mantel-Haenszel method for variance estimation was used for the qualitative data. Risk ratio (RR) calculation was used to evaluate the impact of ECMO on the last reported mortality, treatment failure and need for RRT. Means were calculated from medians using the Wan method.^(14)^ For the quantitative pooled analysis, the DerSimonian and Laird method was chosen. We performed the analyses with free source R software (www.r-project.org) 3.4.1 with the meta package.^(15)^

## RESULTS

**Figure 1:**
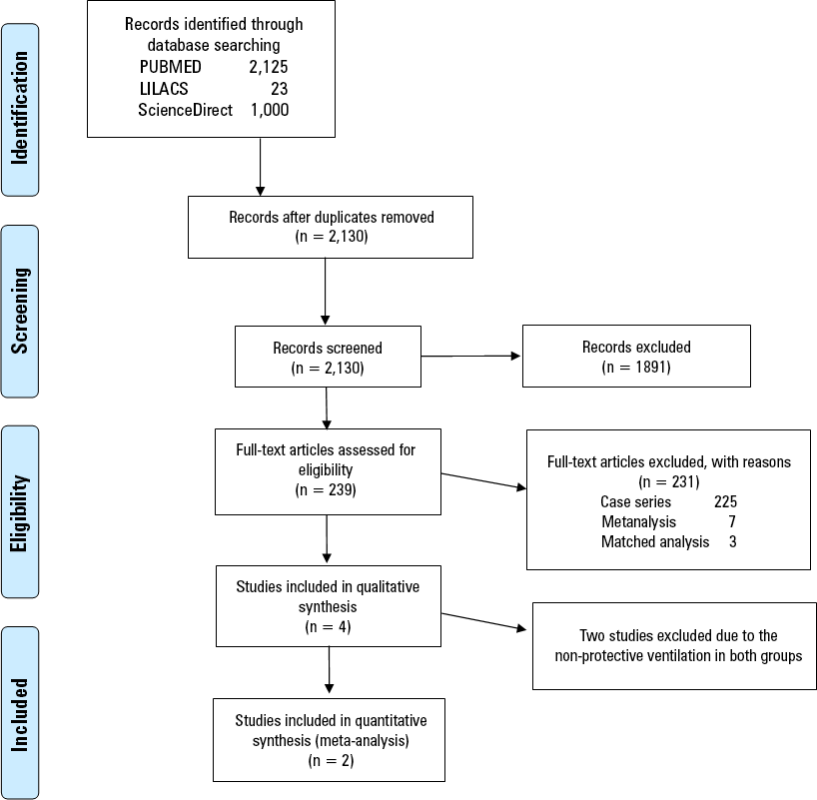
PRISMA flowchart of the systematic literature review.

**Figure 2:**
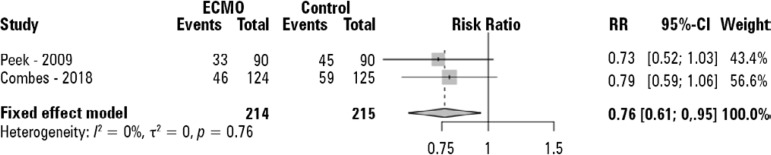
Pooled analysis of the last reported mortality. ECMO - extracorporeal membrane oxygenation; RR - risk ratio; 95%CI - 95% confidence interval. Peek et al.^(6)^ reported the six-month mortality. Combes et al.^(9)^ reported 90-day mortality. The pooled estimate was calculated with the Mantel-Haenszel model.

Our search strategy retrieved two studies that fulfilled all inclusion and exclusion criteria. Figure 1 shows the PRISMA flowchart for the systematic literature review. Both trials had a low risk of bias, and the main characteristics of these studies are described in table 1. A total of 429 patients were included in the analysis; 214 were randomized to receive ECMO support; and 215 were randomized to receive conventional protective mechanical ventilation. Seventy-nine out of 214 patients (37%) randomized to the ECMO group and 104 out of 215 patients (48%) randomized to the control group died at the last reported mortality analysis (90 and 180 days) with a RR of 0.76 (95% confidence interval - 95%CI 0.61 - 0.95) in favor of the ECMO group (Figure 2).

**Table 1:** Analyzed studies' main characteristics

Study analyzed	Peek et al.,^(6)^	Combes et al.,^(9)^
Study type	Randomized multicenter controlled trial	Randomized multicenter controlled trial
Sample size	ECMO group: 90 patients Control group: 90 patients	ECMO group: 124 patients Control group: 125 patients
Total of ECMO supported patients	ECMO group: 68 Control group: none	ECMO group: 124 Control group: 35
Groups crossover	None	35 (28%) patients from control to ECMO group after 6.5 ± 9.7 days
Age	ECMO group: 40 ± 13 years-old Control group: 40 ± 13 years-old	ECMO group: 52 ± 14 years-old Control group: 54 ± 13 years-old
Patient illness severity	APACHE II: ECMO group: 20 ± 6 Control group: 20 ± 6	SAPS II: ECMO group: 42 ± 15 Control group: 41 ± 14
Etiology of respiratory failure	Pneumonia 60% Other ARDS 28% Trauma 7% Others 5%	Pneumonia 65% Aspiration 10% Sepsis 3% Pancreatitis 3%
Enrollment criteria	Murray score ≥ 3 OR Uncompensated hypercapnia with pH < 7.2	PaO_2_/FiO_2_ < 50 for > 3 hours despite rescue maneuvers and protective ventilation PaO_2_/FiO_2_ < 80 for > 6 hours despite rescue maneuvers and protective ventilation pH < 7.25 and PaCO_2_ > 60mmHg for > 6 hours despite maximal minute ventilation respecting RR < 35 BPM, Pplat < 32cmH_2_O and 4 - 8mL/kg of tidal volume
Exclusion criteria	Ppeak > 30cmH_2_O or high FiO_2_ > 0.8 ventilation for more than 7 days; intracranial bleeding; contraindication for heparinization; or any contraindication for continuation of active treatment.	Age < 18 years; mechanical ventilation ≥ 7 days; pregnancy; BMI > 45kg/m^2^; chronic respiratory or cardiac failure; heparin-induced thrombocytopenia; life expectancy < 5 years; SAPS II > 90; coma after cardiac arrest; irreversible neurologic injury; withhold or withdraw life-sustaining therapies; or difficulty in vascular access
Hypoxemia as enrolment reason	ECMO group: 85 (94%) patients Control group: 87 (97%) patients	ECMO group: 99 (80%) patients Control group: 105 (84%) patients
Hypercapnia as enrolment reason	ECMO group: 5 (5%) patients Control group: 3 (3%) patients	ECMO group: 25 (20%) patients Control group: 20 (16) patients
Murray's score at enrolment	ECMO group: 3.5 ± 0.6 Control group: 3.4 ± 0.3	ECMO group: 3.3 ± 0.4 Control group: 3.3 ± 0.4
P/F ratio at enrolment	ECMO group: 76 ± 30 Control group: 75 ± 36	ECMO group: 73 ± 30 Control group: 72 ± 24
Continuous or intermittent RRT needed	ECMO group: 72 (80%) patients Control group: 76 (84%) patients	ECMO group: 65 (52%) patients Control group: 81 (64%) patients
Bypass configuration	Venous-venous: 68 patients	Venous-venous: 152 patients Venous-arterial ECPR: 6 patients Venous-arterial non-ECPR: 1 patient
ECMO membrane	Polymethylpentene	Polymethylpentene
ECMO blood pump	Peristaltic	Centrifugal
Interhospital transportation	Without ECMO support	On pump
Time from intubation to randomization	ECMO group: 35 [17,105] hours Control group: 37 [16,102] hours	ECMO group: 34 [15,89] hours Control group: 34 [17,100] hours
Time on ECMO support	9 [6,16] days	15 ± 13 days
Mechanical ventilation	ECMO group: PEEP 10 - 15cmH_2_O, Ppeak 20 - 25cmH_2_O, FiO_2_ 0.3, and respiratory rate 10 breaths/minute Control group: tidal volume 4 - 8mL/kg and Pplat < 30cmH_2_O	ECMO group: PEEP ≥ 10cmH_2_O, tidal volume to Pplat ≤ 24cmH_2_O, RR 10 - 30 BPM and FiO_2_ 0.3 - 0.5 Control group: Increased recruitment strategy as in express trial
ICU-LOS	ECMO group: 24 [13,41] days Control group: 13 [11,16] days	ECMO group: 23 [13,34] days Control group: 18 [8,13] days
Hospital-LOS	ECMO group: 35 [16,74] days Control group: 17 [5,45] days	ECMO group: 36 [19,48] days Control group: 18 [5,43] days
Last reported deaths in ECMO group	Six months: 33 (37%)	90 days: 46 (37%)
Last reported deaths in control group	Six months: 44 (49%)	90 days: 59 (47%)

ECMO - extracorporeal membrane oxygenation; APACHE II - Acute Physiology and Chronic Health Evaluation II; SAPS II - Simplified Acute Physiology Score II; ARDS - acute respiratory distress syndrome; PaO_2_ - partial pressure of oxygen; FiO_2_ - inspired oxygen fraction; RR - respiratory rate; Pplat - plateau pressure; Ppeak - peak pressure; BMI -body mass index; RRT - renal replacement therapy; ECPR - extracorporeal cardiopulmonary resuscitation; PEEP - positive end-expiratory pressure; ICU - Intensive care unit; LOS - length-of-stay; BPM - breaths per minute.

In the treatment failure analysis, 71 out of 192 (46%) patients randomized to the ECMO group died, while 127 out of 237 (54%) patients died or switched to ECMO (crossover) in the control group. Figure 1S (Supplementary material) shows the treatment failure pooled analysis, with a RR of 0.68 (95%CI 0.55 - 0.85] in favor of the ECMO group.

In the per protocol analysis, only 68 out of 90 patients randomized to the ECMO group (76%) received ECMO support in the CESAR trial.^(6)^ In contrast, in the EOLIA trial,^(9)^ 35 patients from the control group required rescue ECMO support, for a total of 159 patients supported with ECMO. Of all ECMO-supported patients, 91 out of 227 patients (40%) died, while 92 out of 202 patients (46%) without ECMO support died. We show this pooled analysis in figure 2S (Supplementary material), with a neutral RR result of 0.88 (95%CI 0.70 - 1.11).

**Figure 3:**
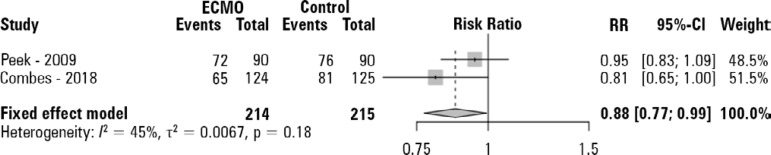
Need for renal replacement therapy pooled analysis of the two retrieved studies. ECMO - extracorporeal membrane oxygenation; RR - risk ratio; 95%CI - 95% confidence interval. The pooled estimate was calculated with the Mantel-Haenszel model.

**Figure 4:**
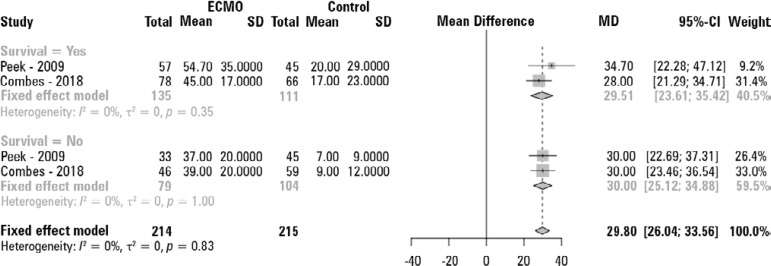
Pooled analysis of in-hospital length of stay. ECMO - extracorporeal membrane oxygenation; SD - standard deviation; MD - mean difference; 95%CI - 95% confidence interval. The pooled mean and 95% confidence interval estimate were calculated with the DerSimonian and Laird model.

Figure 3 shows the need for RRT, with a RR of 0.88 (95%CI 0.77 - 0.99) in favor of ECMO. Figure 3S (Supplementary material) and figure 4 show the ICU and hospital LOS pooled analysis, with substantial increases in the ICU (mean difference [95%CI - 14.84 (12.49 - 17.18) days] and hospital [29.80 (26.04 - 33.56) days] LOS in the ECMO group.

## DISCUSSION

Our results shows that respiratory ECMO support may improve survival in select severe ARDS patients with a RR of 0.76 (95%CI 0.61 - 0.95). Furthermore, the impact of respiratory ECMO support on patient outcomes may be even more striking if treatment failure is used as an endpoint. Considering that ARDS is still a condition associated with a high mortality rate^(16)^ and that protective mechanical ventilation is one of the cornerstones of the methods used to reduce mortality in ARDS patients,^(17)^ it seems reasonable that the adoption of (ultra)protective mechanical ventilation in association with ECMO support may reduce mortality in this population.

Although both previously published randomized controlled trials of ECMO support in association with protective mechanical ventilation have shown conflicting conclusions, it is important to notice that some limitations of each trial design may be responsible for these results. In the CESAR trial, (ultra)protective ventilation was able to improve the six-month outcomes of severe ARDS patients.^(6)^ However, the lack of a standardized protocol for protective mechanical ventilation in the control group, as well as the transfer of patients to a specialized referral center in the ECMO group, may suggest that a higher than usual mortality rate in the control group instead of an increase in the survival in the ECMO group could explain the results.

On the other hand, the recently published EOLIA trial showed no survival benefits with ECMO support in comparison to protective mechanical ventilation alone. However, it should be highlighted that the trial sample size calculation was based on a 20% reduction in the absolute risk of death. Therefore, this large reduction in the absolute risk of death in association with a triangular boundary design to stop the trial probably led to early cessation of the trial with underpowered, neutral results, in which the Kaplan-Meier curve showed results in favor of ECMO support, with a p value for the log rank test of 0.07. In addition, the presence of a crossover rate of 28% from the control to the ECMO group raises concerns regarding the validity of the trial. To overcome this limitation, we designed the analysis of death and crossover to ECMO support in the control group as treatment failure, which showed an improvement in survival rates in favor of ECMO support. One should note that 20 out of 35 (57%) patients in the control group who received ECMO died. This high mortality rate may be attributed to late initiation of ECMO support after randomization (6.5 ± 9.7 days), which occurred 34 (95%CI 15 - 100) hours after intubation. In addition, six patients underwent venous-arterial bypass during cardiac arrest resuscitation. Initially, both cardiac arrest and the need for ECMO support after several days of mechanical ventilation were predefined exclusion criteria in the EOLIA trial due to poor prognosis.^(9,18)^

The reduction in the need for RRT in ECMO-supported patients corroborates the findings of previous reports that severe hypoxemia may increase renal arterial impedance.^(19)^ Moreover, the (ultra)protective mechanical ventilation during ECMO support may have reduced extrapulmonary organ damage and acute kidney injury due to reduced biotrauma.^(20)^ Finally, the use of ECMO support resulted in the survival of critically ill patients who would not survive otherwise, with a consequent increase in ICU and hospital LOSs.

### Limitations

Our study has several limitations. First, the small number of randomized controlled trials surely affects the validity of a meta-analysis. However, it is important to note that the execution of large trials with the use of ECMO support is costly and demands a substantial effort, which is very unlikely to happen again. In this scenario, a metanalysis of available data seems more feasible and important to clinical practice. Second, it is not possible to blind physicians to ECMO support, and therefore, some performance bias may be present in the evaluated trials. In addition, the high rates of crossover in both trials may have also affected our results. Third, our search strategy was limited to Medline, Lilacs, and Science Direct, and we did not expand our search into the so-called gray literature. However, it is highly unlikely that a randomized controlled trial for ECMO support fulfilling our inclusion criteria would be missed. Finally, although patients in both trials represent a very homogeneous ARDS population, the strategy of transportation of patients and ECMO initiation differed substantially between both trials, possibly influencing our results.

## CONCLUSION

Extracorporeal membrane oxygenation support is associated with a reduced mortality rate and a reduced need for renal replacement therapy in severe acute respiratory distress syndrome patients. As a drawback, the intensive care unit and hospital length of stay are markedly higher in patients who receive respiratory extracorporeal membrane oxygenation support compared to those who receive conventional mechanical ventilation.
