# Supplementary material for: Extracorporeal membrane oxygenation for severe acute respiratory distress syndrome in adult patients: a systematic review and meta-analysis
Source: Rev Bras Ter Intensiva. 2019 Oct-Dec;31(4):548–54. doi: 10.5935/0103-507X.20190077 (PMC7008998; doi:10.5935/0103-507X.20190077)
Supplement: Supplementary file 1 [file rbti-31-04-0548-suppl1.pdf]

Extracorporeal membrane oxygenation for severe acute respiratory distress syndrome in adult patients: a systematic review and meta-analysis

Oxigenação por membrana extracorpórea para síndrome do desconforto respiratório agudo grave em pacientes adultos: revisão sistemática e metanálise

Pedro Vitale Mendes[1], Livia Maria Garcia Melro[1], Ho Yeh Li[1], Daniel Joelsons[1], Rogerio Zigaib[1], José Mauro da Fonseca Pestana Ribeiro[1], Bruno Adler Maccagnan Pinheiro Besen[1], Marcelo Park[1]

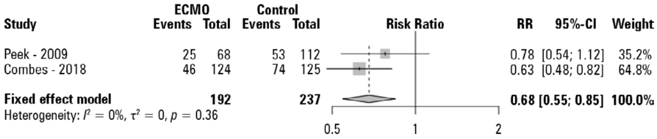

Figure 1S - Pooled analysis of the last reported mortality of the two retrieved studies, taking into account only patients who received extracorporeal membrane oxygenation support in the CESAR trial and considering deceased and crossover patients in the control group of the EOLIA study as treatment failure. ECMO - extracorporeal membrane oxygenation; RR - risk ratio; 95%CI - 95% confidence interval. Peek et al.[6] reported six-month mortality. Combes et al.[9] reported 90-day mortality. The pooled estimate was calculated with the Mantel-Haenszel model.

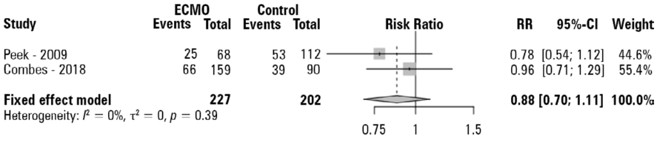

Figure 2S - Pooled analysis of the last reported mortality of the two retrieved studies, taking into account separately all patients who received ECMO support at any time and all patients who never received ECMO support. ECMO - extracorporeal membrane oxygenation; RR - risk ratio; 95%CI - 95% confidence interval. Peek et al.[6] reported six-month mortality. Combes et al.[9] reported 90-day mortality. The pooled estimate was calculated with the Mantel-Haenszel model.

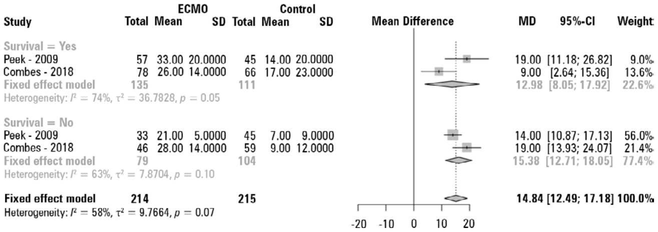

Figure 3S - Pooled analysis of intensive care unit length of stay. ECMO - extracorporeal membrane oxygenation; MD - mean difference; 95%CI - 95% confidence interval; SD - standard deviation. The pooled mean and 95% confidence interval estimate were calculated with the DerSimonian and Laird model.

Table 1S - Risk of bias of the studies analyzed

	Peek et al.[6]	Combes et al.[9]
Selection bias	Low risk	Low risk
Reporting bias	Low risk	Low risk
Performance bias	Low risk	Low risk
Detection bias	Low risk	Low risk
Attrition bias	Low risk	Low risk
Other bias	Low risk	Low risk
